# Treatment of Scarlet Fever

**Published:** 1909-04

**Authors:** 


### Treatment of Scarlet Fever

Professor E. Grawitz, (*Therap. Monatsh.*, December, 1908,) says that in view of the important role which the kidneys play in combating infection, prognosis at once becomes unfavorable when a nephritis hinders elimination of toxic products. The course of scarlatina or diphtheria, as can not be too strongly urged, is most unfavorably influenced when, in local treatment of the larynx, some of the antiseptic is swallowed or absorbed, and thus notorious renal irritants get into the system. The integrity of this most important excretory organ must be preserved. And therefore prophylaxis with urotropin, which acts mildly as a disinfectant in the urinary passages, is very commendable, as his success in scarlatinal therapy shows. But also in septic infections of different origin this renal prophylaxis is entirely rational.

Small children with scarlatina get, on admission, 0.25 gm. (4 grs.) urotropin thrice daily; large ones and adults get 0.5 gm. (7½ grs.) thrice daily, for four successive days. After a four days' interval, it is resumed for another four days, again withdrawn for the same period, and a final four days' course given. Albuminuria on reception is no counterindication; rather does it often disappear under urotropin.

During over four years, in more than 500 cases, his success from this procedure is so brilliant and unequivocal that he gives it, to a large extent, the credit for his low mortality figures. In patients admitted with non-affected kidneys, he has seen but 5 to 6 per cent. of renal irritation and inflammation, and most of these were only transient. Not one patient was left with a chronic nephritis. What is especially convincing, not one case of uremia was observed. Formerly, in the midst of a smooth recovery, a hemorrhagic nephritis with chills and uremia would suddenly set in. This severe complication has not been seen at all in recent years. Even patients admitted only on the third day of the disease, with renal irritation, extremely rarely suffered the severe sequels of nephritis. Phlebotomy he has in recent years used only in such isolated cases as were turned over to him because of renal complications.

